# Nucleosomes and their complexes in the cryoEM era: Trends and limitations

**DOI:** 10.3389/fmolb.2022.1070489

**Published:** 2022-11-24

**Authors:** Grigoriy A. Armeev, Anna K. Gribkova, Alexey K. Shaytan

**Affiliations:** ^1^ Department of Biology, Lomonosov Moscow State University, Moscow, Russia; ^2^ Department of Computer Science, HSE University, Moscow, Russia

**Keywords:** chromatin proteins, protein-protein interactions (PPI), structural datasets, nucleosome structure, chromatin structure

## Abstract

Twenty-five years have passed since the appearance of the first atomistic model of the nucleosome structure, and since then the number of new structures has gradually increased. With the advent of cryo-microscopy, the rate of accumulation of models has increased significantly. New structures are emerging with different histone variants and a variety of proteins that bind to nucleosomes. At the moment, there are more than four hundred structures containing nucleosomes in the Protein Data Bank. Many of these structures represent similar complexes, others differ in composition, conformation and quality. In this perspective, we investigate the diversity of known nucleosome structures, analyze data and model quality, variations in histone/DNA content of nucleosomes and spectrum of their interactors. We outline those parts of the nucleosome “structurome” that are already explored and those awaiting further exploration.

## Introduction

**FIGURE 1:**
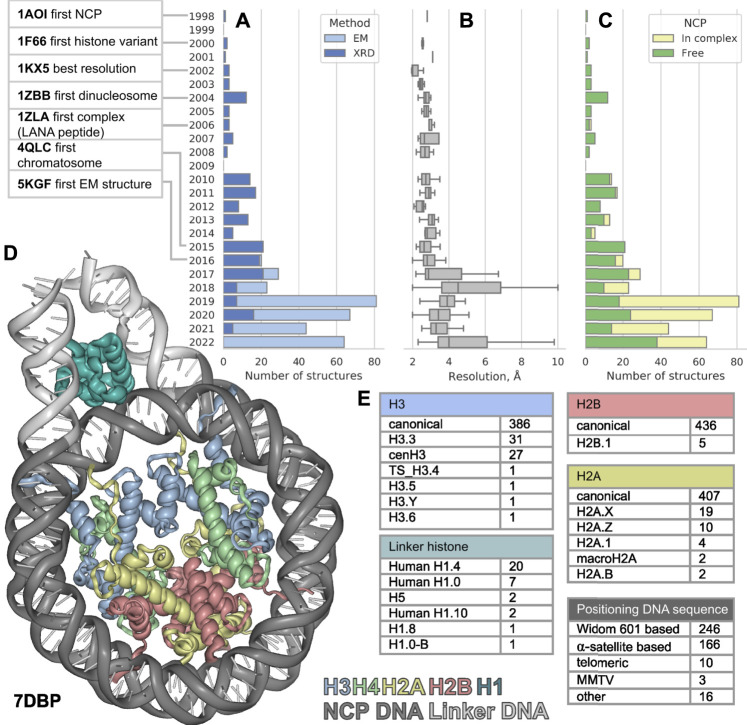
The distribution of nucleosome-containing structures published per year since 1998 **(A)** subdivided by acquisition method, **(B)** structure resolution distribution **(C)** subdivided by type of structure: Free nucleosomes or nucleosomes in complex with non-histone proteins. **(D)** Visualization of nucleosome in complex with linker histone, linker DNA region is shown in light gray. **(E)** Variety of nucleosome components among the known structures. The PDB IDs for nucleosome component variants are presented in Supplementary Table S1.

Chromatin combines a high degree of compaction with the ability to access the DNA for transcription. Chromatin is subject to constant rearrangements and does not have a permanent structure. At the basic level of packaging, eukaryotic DNA forms complexes with proteins - nucleosomes. Each nucleosome carries about 200 DNA b.p. only 145–147 b.p. of which are associated with proteins constituting nucleosome core particle (NCP), the remaining 50 bp are called linkers, as they connect neighboring NCPs (Figure 1D). The protein core of the nucleosome comprises four types of histones, which in pairs form histone dimers H3-H4 and H2A-H2B. A complete nucleosome contains two dimers of each type, however, histones can form smaller subnucleosomal particles (Zlatanova et al., 2009; Armeev et al., 2019). There is a separate type of histone, the linker histone also called H1 histone (or H5 in avian species), which binds to the nucleosome to form a chromatosome. Chromatosomes are more stable than nucleosomes and allow chromatin to be packed more tightly (Fan et al., 2003). Histone genes are divided into replication-dependent (canonical histones) and replication independent (histone variants), which may be involved in specific nuclear processes (Seal et al., 2022). Nucleosomes are also the target for an extensive family of non-histone chromatin proteins. Such proteins specifically interact with nucleosomes to perform a wide range of functions, from kinetochore formation during DNA replication to regulation of transcription and DNA repair.

The first atomic NCP structure was obtained in 1997 (deposited to PDB in 1998) by X-ray diffraction (XRD) (Luger, 1997). In the past 25 years after the first one, 441 new nucleosome containing structures were released (as of 20 September 2022), nearly half of them were released in the last 5 years. Such burst could be explained by recent rapid progress in cryo-electron microscopy (cryoEM). Numerous structures of nucleosomes with histone variants have appeared, as well as structures of complexes with other chromatin proteins. However, this flow of structures makes it difficult to keep pace and see all available information about chromatin structure at its basis. In this work, we examine a set of all currently available structures of nucleosomes and their complexes. We evaluate the diversity of nucleosome components and proteins associated with them.

## Resolution and quality of known nucleosome structures

Since the first structure released in 1997 until 2015 all 113 structures of nucleosomes in PDB were obtained by XRD. The yearly median resolution of those structures was better than 3 Å, and is sufficient for protein backbone and large side chains position determination. The best structure in terms of resolution was released in 2002 (Davey et al., 2002). The structure presented a complete atomic picture of a nucleosome assembled on a modified human α-satellite DNA sequence, together with bound water molecules and partially resolved histone tails. In 2016 the first structure of nucleosome obtained by cryoEM was published with resolution of 4.5 Å (Wilson et al., 2016, 1) and ever since 253 structures were obtained with cryoEM and 188—with XRD (Figure 1A). As a result, the per-year median resolution of nucleosome structures has decreased significantly, sometimes worse than 4 Å. (Figure 1B). However, the criteria for determining resolution may be different between cryoEM structures. The resolution in cryoEM structures is unevenly distributed and could be significantly worse in the regions of interest. Despite a general decrease in the resolution of structures, the quality of the models is growing, judging by the clash-score, rotamer-outliers (Supplementary Figures S1A,B). Such a trend suggests that the models might be biased towards optimal molecular geometry rather than experimental structure. The local resolution is usually lower in highly mobile regions, such as DNA in nucleosomes, flexible histone tails and interacting proteins. CryoEM resolution of nucleosome-containing structures is steadily improving (Supplementary Figure S1C), reaching the XRD level of detail for some structures. We expect an improvement of nucleosome structure resolutions in the future by establishing consistent protocols as the method matures. However, for EM structures it is always important to assess the local resolution of electron density maps to separate the confident model areas from vaguely defined zones.

## Variety of DNA and histones in nucleosome structures

It is known that the structure of nucleosome depends on the DNA sequence. There are three main groups of nucleosomes containing such sequences present in PDB. The largest group contains “Widom 601” sequence (Lowary and Widom, 1998) and its derivatives—246 structures (this is an artificially created high affinity nucleosome positioning sequence). The second sequence group is α-satellite sequences and their derivatives—166 structures (α-satellite sequence is a natural sequence from human centromeric chromosome region). Most of the nucleosome structures in this group contain 146 bp of DNA (90 structures), 26 structures contain shortened 145 bp DNA α-satellite sequence and 26 structures contain longer 147 bp DNA sequence. The third sequence group contains 10 structures with telomeric sequence and several other sequences (cen3 DNA, MMTV, DNA1, sat2 and Human-D02, see Supplementary Table S1). Despite the fact that hundreds of different nucleosome structures are stored in PDB, their DNA sequences belong to a rather limited set. It is clear that increase in the variety of different DNA types in nucleosome structures is vital for understanding the nucleosome positioning. The median resolution across all nucleosomal structures is 3.3 Å, since DNA in nucleosomes is more mobile than histones the effective resolution in DNA areas is lower. Thus, half of nucleosome structures likely inherit DNA geometry from other high-resolution structures used as templates.

Histone variants provide some specific functions, for example participate in various biological processes such as gene expression, DNA replication and DNA damage repair. Even a few amino acid substitutions in histone variants may alter nucleosome dynamics and stability (Dai et al., 2021). Therefore, the NCP structures with different histone sequences may shed light on the physical nature of the specific biological processes in which histone variants are involved.

We employed histone variant annotation from HistoneDB 2.0 (Draizen et al., 2016) to assess the distribution of histone variants in NCPs across the PDB (Figure 1E). H3 is the most represented histone type in nucleosome structures; PDB contains the following variants: H3.3, cenH3, TS H3.4, H3.5, H3.6, H3.Y. There are structures with H2A variants H2A.1, H2A.B, H2A.X, H2A.Z and macroH2A. However, macroH2A is present in truncated form (around 107 out of 370 amino acids). Variants H2A.L, H2A.P, H2A.W are still missing in PDB. The latter variant H2A.W is a plant specific histone with a unique SPKK motif in the C-terminal tail, whose implications on nucleosome structure are yet to be characterized. There is only one H2B variant present in PDB-deposited nucleosome structures - H2B.1, while other histone variants, which are involved in spermiogenesis (H2B.W, sperm H2B and subH2B) are not available.

The effect of histone variants on the nucleosome structure and dynamics is one of the major challenges in structural studies. However, even canonical histones vary between species. Most of the nucleosomes contain human and model organisms histones: *Homo sapiens* (219 structures), *Xenopus laevis* (172), *Saccharomyces cerevisiae* (12), *Drosophila melanogaster* (8), *Mus musculus* (7). But there are structures of nucleosomes with histones of methylotrophic yeast *Komagataella pastoris* which are used for protein production (PDB ID: 7WLR) (Fukushima et al., 2022), nucleosome of pathogenic unicellular eukaryotic parasite *Giardia lamblia* (PDB ID: 7D69) (Sato et al., 2021) and human nucleosome with incorporation of protozoan parasite *Leishmania* histone H3 (PDB ID: 6KXV) (Dacher et al., 2019). The first studies of archaea histones date to 1990 (Sandman et al., 1990), however the structure of histone-based archaeal chromatin was resolved only recently. There is only one structure of archaeal so-called “hypernucleosomes” from Methanothermus fervidus (PDB ID: 5T5K) (Mattiroli et al., 2017), where DNA is wrapped around an “endless” histone-protein core. Interestingly, several viruses encode their own histones, while other typo viruses can even incorporate host eukaryotic histones into their nucleosomes. The first examples of viral nucleosomes of Marseilleviridae (giant viruses that infect amoebae) were resolved in 2021 (PDB IDs: 7LV8, 7LV9, 7N8N) (Liu et al., 2021a; Valencia-Sánchez et al., 2021). Marseilleviridae histones are present in doublets (H4 is fused to H3 and H2B is fused to H2A) and form particles similar to eukaryotic “canonical” nucleosomes, though less stable and contain only 120 bp of DNA.

## Higher order chromatin structures

The linker histone H1 binds to the nucleosome near the DNA entry-exit point, thus forming a chromatosome. Currently, there are 33 structures containing linker histone, predominantly human H1.4 variant (20 structures). Also, nucleosome structures contain human H1.0 and H1.10 variants, chicken H.5 and one structure from *Xenopus* with H1.0 and H1.8. Variants scH1, TS H1.6, TS H1.7, TS H1.9 are not present in structure databases now (Figure 1C).

The geometry of nucleosome fibers changes in the presence of linker histone (Routh et al., 2008). The PDB contains 24 models containing parts of such fibers, both with and without a linker histone. Here, we assessed the available models (bioassemblies) and counted nucleosomes located on continuous double-stranded DNA segments. The majority of supranucleosomal structures contain two nucleosomes (11 structures), 8 structures contain three nucleosomes and 5 structures contain four nucleosomes and one structure contain six nucleosomes (PDB ID: 6HKT). Even larger fibers containing 12 and 24 nucleosomes were studied (Song et al., 2014), unfortunately the models were not deposited to the PDB. The studies of larger fibers are of great importance, though much more complex due to high flexibility of their structure.

## Nucleosome complexes

**FIGURE 2:**
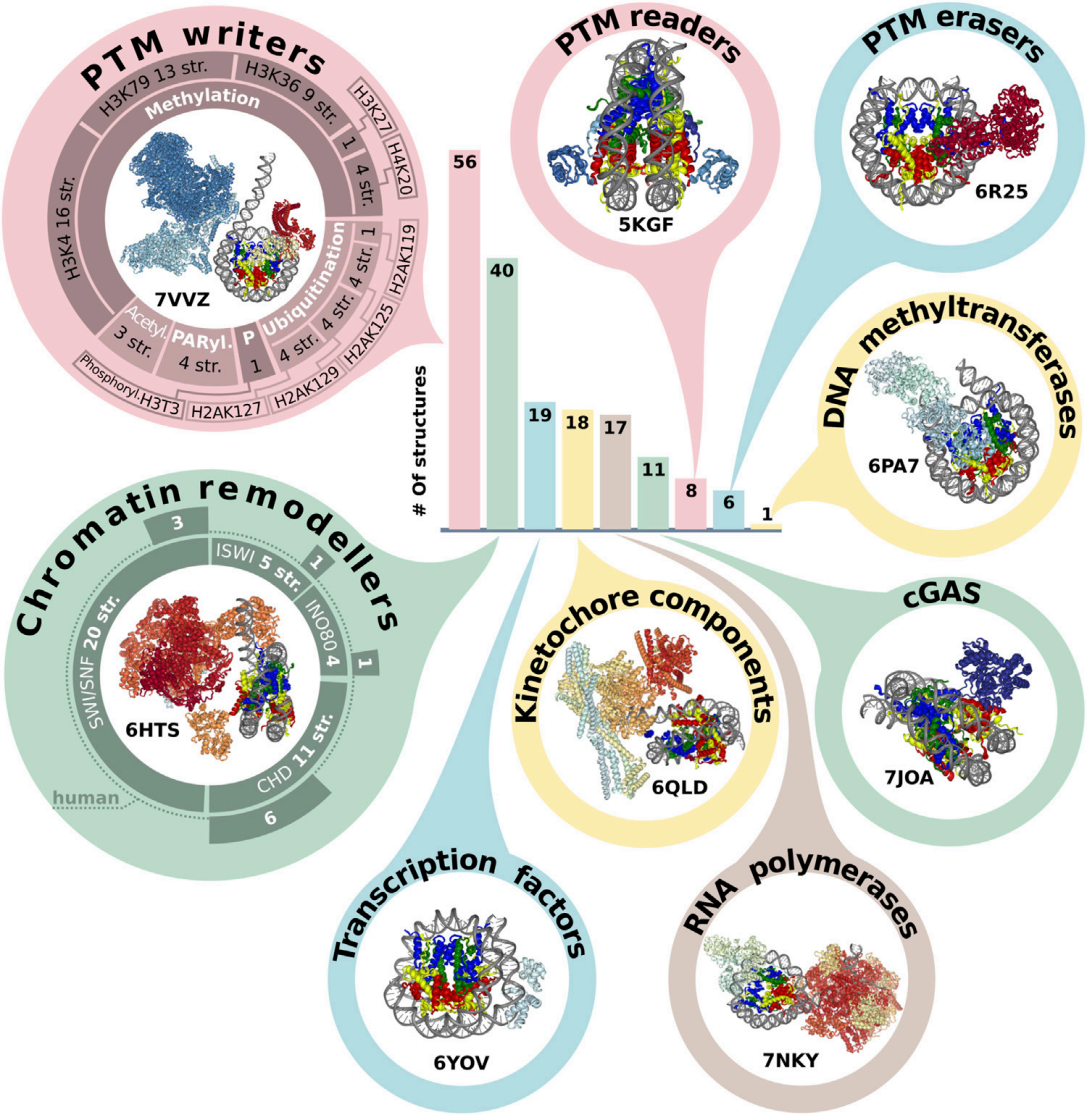
Representation of NCP in protein complexes. The bar plot indicates the number of structures annotated by the molecular functions (see text). The structures of class representatives are depicted in circles with PDB IDs. The panel around PTM (post-translational modification) writers represents a number of structures associated with modification (methylation, ubiquitination, acetylation, PARylation and phosphorylation) of specific sites. The panel around chromatin remodelers represents the number of structures in the chromatin remodeler family, which are additionally subdivided into human and non-human complexes. The infographic does not include the following categories: Histone chaperone (three structures), Histone exchange (two structures), DNA integration (two structures). The PDB IDs for each protein category are presented in Supplementary Table S2.

A plethora of different non-histone proteins interact with nucleosomes to regulate nuclear processes. It is important to study the mechanism of such interactions, as they provide an insight into the fundamental basis of genome regulation. There are 193 structures that contain NCPs in complex with other proteins. The first six structures were resolved by XRD in the period from 2006 to 2013. The interactors resolved in the first nucleosome complexes were relatively small peptides or proteins - a fragment of Kaposi’s sarcoma herpesvirus LANA protein (PDB ID: 1ZLA), regulator of chromosome condensation factor (RCC1, PDB ID: 3MVD), regulatory protein SIR3 (PDB IDs: 3TU4, 4JJN, 4KUD, 4LD9). Since 2016 the number of complexes has rapidly increased, most of them are obtained with cryoEM but have relatively low resolution (Supplementary Figure S1D). The years 2019 and 2020 were the most fruitful in terms of published nucleosome complexes - 60 and 38, respectively (*versus* 21 and 29 for single nucleosomes). Molecular weight of NCP interactors spans from 0.4 to 687 kDa with 53 kDa median. The heaviest complex is the histone acetyltransferase NuA4 complex, shown in Figure 2 (PDB ID: 7VVZ).

There are 215 structural non-histone nucleosome interacting partners found in PDB. The majority of proteins belong to *Saccharomyces cerevisiae* proteins (96 proteins in 54 structures) and human proteins (78 proteins in 102 structures). Interestingly, there are only 15 nucleosomes containing *Saccharomyces* histones. The half of interactor proteins (94 out of 215) are present only in a single structure, and 89 are present in two or more structures. Not all protein sequences are resolved in the structures, but the length of the resolved protein fragments steadily increases (Supplementary Figure S2A).

To assess the functional diversity of NCP complexes with non-histone interactors we designed a simplified protein classification scheme [based on UniProt protein annotation (The UniProt Consortium, 2021) and Gene ontology (Ashburner et al., 2000; The Gene Ontology Consortium, 2021)]. This scheme describes Biological Process, Molecular function and association of structure with histone post-translational modifications (PTM). According to this scheme, the majority of NCP complexes with non-histone interactors belong to the following biological processes: Transcription associated (112 structures), DNA repair (34) and Cell division (24), (Supplementary Figure S2B).

There are five most represented molecular functional protein groups in PDB by our classification: PTM writers (56 structures), Chromatin remodelers (40), Transcription factors (19), RNA polymerases (17) and complexes with Cyclic GMP-AMP synthase (guanosine monophosphate-adenosine monophosphate synthase) (noted as cGAS) (11) (Figure 2). There are 17 structures of NCP with RNA polymerase. However, there are no human RNA polymerases, even in complexes with human histones. All interactors in this group are from *Saccharomyces cerevisiae* or *Komagataella phaffii*.

We also analyzed the representation of human chromatin proteins of different functional classes in the structures of the complexes, Supplementary Figure S2C. The most structurally studied class is kinetochore components (70%). In other classes (Chromatin remodelers, PTM erasers, PTM readers, PTM writers) the range of coverage is 2%–18%.

One of the epigenetics regulation layers is histone post translational modifications, which can alter nucleosome structural dynamics and regulate DNA accessibility (Bernier et al., 2015; Brehove et al., 2015; Kim et al., 2015). We analyzed NCP complexes’ structures for association with histone PTMs and revealed that one-third of NCP complexes is associated with methylation and one-seventh with ubiquitination, Figure 2. Human most studied methylation sites include H3K4, H3K9, H3K27, H3K36, H3K79, H4K20, H3R8, H4R3 [annotated at HISTome 2 (Shah et al., 2020)]. The largest number of structures is associated with methylation of H3K4 (16 structures) predominantly by COMPASS complex, this mark indicates active gene promoters. H3K79 methylation—one of marks of transcription regulation and DNA damage response is presented in 13 structures and the mark of active chromatin H3K36—in 9 structures. Currently, 8 out of 30 human histone lysine methyltransferases are in the structures of NCP complexes and there are no structures of NCP in complex with human methyltransferases for H3K9 and H4R3 sites. First structures with acetylation machinery appeared in 2022 (PDBs: 7W9V, 7VVZ, 7VVU). Less than 10 structures are linked with writing or reading PTMs from the following sites: phosphorylation of H3T3; methylation of H3K27, H4K20; demethylation of H3K4, H3K9 and ubiquitination of H2AK15, H2A119, H2AK127, H2AK125, H2AK129, H2BK120 Figure 2, Supplementary Table S2.

Another key functional class of chromatin complexes is chromatin remodelers, which modify the composition or location of nucleosomes using the energy from ATP hydrolysis. The resolution revolution in cryoEM contributed to the emergence of chromatin remodeler typo complexes in 2017. Chromatin remodelers are divided into four families, the most represented in complexes with nucleosomes is SWI/SNF family (29 structures), followed by CHD (13), INO80 (6) and ISWI (5) (Supplementary Table S2). Thus, the discovered structures cover all known remodeler families, and even if the resolution is low, it is possible to obtain some mechanistic insights about protein-DNA interactions. The next stage of nucleosome complexes research might be expanding the range of proteins interacting with nucleosomes to other DNA interacting proteins. For example, there are currently no structural details about the interplay between nucleosome and CRISPR-Cas systems, which are potential candidates for novel therapy approaches and detection systems (Yarrington et al., 2018; Novikov et al., 2021).

## Nucleosome complexes in human pathologies

Nucleosomes play a pivotal role in chromatin maintenance and gene expression regulation. There are well-studied histone mutations which lead to cancers (Nacev et al., 2019; Espiritu et al., 2021). The part of these histone mutations is represented in NCP structures: 23 out of 434 nucleosome structures contain the following histone mutations: Lysine to Glutamine (KQ) Substitutions in the H3 and H4 Histone-Fold Domains (Iwasaki et al., 2011), oncohistone mutations H3.3K36M (yeast and human nucleosomes) (Liu et al., 2021b), H3 and H4 mutated residues located at the protein–DNA interfaces flanking the nucleosomal dyad (Muthurajan et al., 2004).

We analyzed non-histone nucleosome structural interactors that are involved in oncogenesis by OncoKB annotation (Zdobnov et al., 2021). To increase the number of proteins, we also considered human ortholog proteins for non-human proteins from OrthoDB (Zdobnov et al., 2021). As a result, 24 out of 105 human nucleosome interacting genes are annotated in OncoKB (Chakravarty et al., 2017): five oncogenes (EZH2, NSD2, SOX2, DOT1L, SMARCE1) and 18 Tumor Suppressor Gene (ARID1A, BRCA1, DNMT3A, EP300, EZH2, PBRM1, SETD2, SMARCA4, SMARCB1, ARID2, BARD1, KMT2A, KMT2C, SUZ12, PARP1, DNMT3B, SMARCE1, TP53BP1).

Design of novel compounds for epigenetic targets requires detailed structural information of the interaction interface. Meanwhile, there are only 4 non-histone nucleosome interacting proteins which are approved as targets for epigenetic drugs: Poly [ADP-ribose] polymerase 1 (P09874), Poly [ADP-ribose] polymerase 2 (Q9UGN5), DNA (cytosine-5)-methyltransferase 3A (Q9Y6K1), Histone-lysine N-methyltransferase EZH2 (Q15910). To date, one molecule is in clinical trials targeting Lysine-specific histone demethylase 1A (O60341) as provided by ChEMBL database.

## Discussion

Histone proteins are among the most conservative proteins, and indeed from the first glance all nucleosomes in PDB look alike. However, nucleosomes differ in histone variants, PTMs DNA sequences and there are chromatin proteins that alter their structure.

Sequence-dependence of nucleosome assembly still remains a subject of debate. Unfortunately, the variety of PDB-deposited nucleosomal DNA sequences is limited. Moreover, the majority of nucleosome models do not have sufficient resolution and the structure of DNA in such models is usually derived from other high-resolution structures. There is a strong need for systematic research of DNA sequence influence on nucleosome structure and positioning. We believe that such studies currently are limited by cryoEM data analysis pipelines, which limit the ability to reconstruct relatively flexible DNA.

Another under-researched field of nucleosome structural biology is that dealing with the effects of PTMs on nucleosome structure. Given the amount of different PTMs and their combinations, such effects could be feasibly studied *in silico via* full atom molecular dynamics simulation. Besides, the majority of PTMs are located in intrinsically disordered histone tails, thus making computational methods even more suitable for this task.

Large complexes of nucleosomes and parts of chromatin fibers appear in PDB. The size of structures grows together with their conformational flexibility and heterogeneity, making it difficult to apply structural biology methods. XRD structures are influenced by crystal packing effects; cryoEM is not yet capable of reconstructing the structure ensembles of highly flexible complexes. Nevertheless, there are already structures of large complexes that reveal functional roles of nucleosomes, such as nucleosome rearrangements during the RNA polymerase II passage (Kujirai et al., 2018). In order to explore such structures, it is necessary to combine structural biology methods and integrative modeling approaches based on different heterogeneous experimental datasets (Webb et al., 2018).

Despite a certain number of resolved structures of NCP in complexes with other proteins (193 structures), the set of known interactor structures is incomplete. Nonetheless, there are resolved complexes of nucleosomes with all main chromatin protein categories and even all chromatin remodeler families. The latter structures provided crucial molecular details of remodelers action and an interplay between DNA and histone octamer configuration during nucleosome remodeling (Farnung et al., 2017; Liu et al., 2017). However, the number of structures varies considerably between chromatin protein categories and the representation of functional categories is very heterogeneous. Among the PTM writers the majority of resolved nucleosome complex structures are associated with methylation, while acetylation plays an equally important role in the regulation of nuclear processes. The first structures with acetyltransferases were resolved this year. There are NCPs in complex with methyltransferases for extensively studied lysine methylation, however there are no structures with arginine methyltransferases. There is a plethora of possible PTMs including some emerging noncanonical histone modifications, which also need to be studied to reveal a specific mechanism of their functioning.

In this perspective, we considered the diversity of nucleosome structures and their complexes. Nucleosome structural studies are useful not only for fundamental science, but also for a number of applications. For therapeutic applications, non-histone interactors of nucleosomes can serve as specific drug targets, particularly for treatment of cancer. Histone PTMs (Chervona and Costa, 2012; Zhao and Shilatifard, 2019; Lee and Kim, 2022), as well as free nucleosomes in tissues (Fedyuk et al., 2022) can be used as prognostic and predictive biomarkers in oncology diagnostics. On the other hand, it is necessary to study plant nucleosomes, especially those of cultivated plant species. To sum up, there is a need to expand our knowledge of molecular epigenetic processes by building a comprehensive map of nucleosome structural interactions with non-histone partners for fundamental science, medicine and agriculture.

## Data Availability

The original contributions presented in the study are included in the article/Supplementary Material, further inquiries can be directed to the corresponding authors.
