# Supplementary material for: Nucleosomes and their complexes in the cryoEM era: Trends and limitations
Source: Front Mol Biosci. 2022 Nov 24;9:1070489. doi: 10.3389/fmolb.2022.1070489 (PMC9730872; doi:10.3389/fmolb.2022.1070489)
Supplement: Supplementary file 1 [file DataSheet1.PDF]

Supplementary Material

Nucleosomes and their complexes in the cryoEM era: trends and limitations.

Grigoriy A. Armeev [1,†*], Anna K. Gribkova[1†], Alexey K. Shaytan[1,2,*]

†These authors contributed equally to this work and share first authorship

[1]Department of Biology, Lomonosov Moscow State University, 119234 Moscow, Russia.

[2]Department of Computer Science, HSE University, 101000 Moscow, Russia

Supplementary Table 1.PDB IDs for nucleosome containing various histone variants or DNA sequences (from the figure 1).

**Histone or DNA variant**	**PDB IDs**	**Number of structures**
canonical H3	3LZ0,7TN2,3WAA,6PWE,5OXV,7PF4,6M44,6S01,5Z3L,5XF4,6M4H,7CRR,6R93,6NJ9,6T7A,3REH,7ENN,6NOG,6R94,5Z30,7K60,2NQB,6LA2,6W5M,6USJ,7PFA,3AYW,7U52,5GTC,1P3L,7K61,7CCQ,6DZT,6IY3,7BWD,6KW4,5B31,6IRO,7NKX,1ZBB,4WU8,4WU9,1F66,7CRP,6X5A,6Y5E,6JOU,7E9F,7NKY,2PYO,6T9L,6RYR,5JRG,6GEJ,3MGS,7XCR,6K1J,3UTB,6KE9,5OY7,6R91,7OH9,4R8P,6JMA,6L9H,1AOI,6R90,6V2K,6NZO,4KUD,5Z3O,3W98,5B0Z,4ZUX,6X0N,3WA9,1KX4,6K1I,7PFC,6KIV,5XF5,3REJ,3B6F,4JJN,6Y5D,7M1X,4QLC,6PWW,7XPX,7PFE,7V96,5MLU,5CPJ,6FTX,6NE3,6WZ5,6FQ5,3UTA,6O96,6PWV,3LJA,5X0X,3AFA,2NZD,7V9K,2FJ7,7PEZ,7COW,5B0Y,6IY2,7VA4,5CPI,1KX5,6FQ8,5Z3V,6PX1,4J8U,5GSU,1P3F,5F99,5AVB,4XZQ,3KWQ,5ZBX,7LYC,7K6P,7CRQ,7LYA,7EA5,3KXB,4KGC,7PEW,7PET,7K63,7KTQ,5Y0D,3AZF,7U50,6FML,3C1C,6KVD,7V9C,7VVZ,3REK,7E8I,3X1T,4XUJ,7KBF,7TAN,3LZ1,3W96,4Z66,6R1U,6W5N,6M3V,7JO9,6R92,6T7D,1P34,5B1M,2F8N,7V9J,7PF6,3REL,6UPK,5KGF,3X1S,7JZV,6KIZ,3W97,6KXV,3W99,6WZ9,7YQK,7EGP,1P3K,5Z3U,6UGM,1U35,5WCU,6KW3,6ESF,7OHB,5O9G,7MBM,3AZL,3KUY,6YOV,5ONG,5Z23,6JR1,3MGR,7D69,3AZI,7PEU,5HQ2,7V90,6VYP,6PWF,6XJD,6I84,6T79,5NL0,1KX3,6UXW,7KBD,7PFF,6UPL,1EQZ,7DBH,6NQA,5AV5,7XD0,4YS3,3REI,5DNN,3AZE,6JR0,6KIX,1P3A,6KW5,4LD9,7E8D,6T93,4J8W,6GEN,7KBE,7A08,5Y0C,3AZJ,5CP6,1P3I,7PFW,1S32,7PFT,6G0L,6TDA,7SCZ,3X1V,1P3P,6L49,3O62,7PEY,5B1L,1P3B,1M18,6X59,4J8X,6V92,6M4D,7EG6,6VZ4,7V9S,7PFD,5B40,4YM6,7MBN,3MVD,7PFV,6UH5,6VEN,6W5I,6JM9,7BY0,3AZM,7PFU,7SCY,6R8Y,6LAB,1P3G,3LEL,6ESH,6T90,3WKJ,3B6G,6OM3,7BXT,3MNN,7U53,7LYB,6HKT,6JXD,3MGP,5DNM,7CRO,6L9Z,7K5Y,3C1B,7W9V,4J8V,5GT0,6NN6,6HTS,5GSE,3AZH,6K1P,5XF6,7VDT,7NL0,5AY8,7OTQ,7U51,3UT9,6J99,5AV9,6K1K,6IPU,4X23,7CCR,7PF3,7PF0,5XF3,7DBP,7VDV,6L4A,6Z6P,5GT3,1M19,1P3O,6ESI,6IQ4,2CV5,5AV8,6LER,7PEX,7XD1,6R8Z,6PWX,5OMX,3AZK,3MGQ,6T7C,5CPK,5B2J,7Y7I,5ONW,5X0Y,5B24,1M1A,6M4G,7K5X,3AV1,7OHA,6ZHY,7PF2,7K7G,5B2I,5E5A,1ZLA,3X1U,6JYL,7K6Q,4YM5,7JOA,3AZN,7XCT,7VVU,7PEV,5AVC,7OHC,6T7B,6ZHX,6R25,6ESG,7PFX,6PA7,6KIW,6LA9,6FQ6,7SWY,1P3M,6R1T,3AZG,6LE9,7C0M,6KIU,7D1Z,5AV6,6PX3,1ID3,6RYU,3TU4,6LA8,7PF5,7AT8,7E9C	386

H3.3	6IR9,6J4W,6J4Y,6J50,5XM0,7WLR,6R0C,6R25,6LTJ,5X7X,3AV2,6A5R,5B33,3WTP,6J4X,6A5O,6RNY,6INQ,5B32,6A5T,7A08,7DBH,6A5L,5XM1,6J51,7VBM,7EA8,6A5U,6R1U,6J4Z,6A5P	31
cenH3	6SEE,6SEF,6E0P,6MUO,7U46,7U4D,6UPH,6SE6,3WTP,6QLD,7D20,7U47,7K78,6BUZ,6SE0,7YYH,7PII,7ON1,6MUP,3AN2,6TEM,6SEG,6L49,7R5R,6O1D,6C0W,6E0C	27
TS_H3.4	3A6N	1
H3.5	4Z5T	1
H3.Y	5AY8	1
H3.6	5GXQ	1
canonical H4	6E0P,3LZ0,7TN2,3WAA,6UPH,6PWE,5OXV,7PF4,6M44,6S01,5Z3L,5XF4,6M4H,7CRR,6R93,6NJ9,6T7A,3REH,7ENN,6NOG,6R94,5Z30,7K60,2NQB,6LA2,6W5M,6USJ,7PFA,3AYW,7U52,5GTC,1P3L,7U46,7K61,7CCQ,6DZT,6IY3,7BWD,6KW4,5B31,6IRO,7NKX,1ZBB,4WU8,4WU9,1F66,4Z5T,7CRP,6X5A,6Y5E,6JOU,7E9F,7NKY,2PYO,6T9L,6RYR,5JRG,6GEJ,3MGS,7XCR,5X7X,6K1J,3UTB,6KE9,5OY7,6R91,7OH9,4R8P,6JMA,6L9H,1AOI,6R90,6V2K,6NZO,4KUD,5Z3O,3W98,5B0Z,6SEE,4ZUX,6X0N,3WA9,1KX4,6K1I,7PFC,6KIV,5XF5,3REJ,3B6F,6QLD,4JJN,6Y5D,7U47,7M1X,4QLC,6PWW,7XPX,7PFE,7V96,5MLU,5CPJ,6FTX,6NE3,6WZ5,6FQ5,6C0W,3UTA,6O96,6PWV,6R0C,3LJA,6A5R,5X0X,3AFA,2NZD,7V9K,2FJ7,7PEZ,7COW,5B0Y,6IY2,7VA4,6TEM,5CPI,1KX5,6FQ8,5Z3V,6PX1,4J8U,6A5P,5GXQ,5GSU,1P3F,5F99,5AVB,4XZQ,3KWQ,5ZBX,7D20,7LYC,7K6P,7YYH,7ON1,7CRQ,7LYA,7EA5,3KXB,4KGC,7PEW,7PET,7K63,7KTQ,5Y0D,3AZF,5XM0,7U50,6FML,3C1C,6KVD,7V9C,7VVZ,6A5T,3REK,7E8I,3X1T,4XUJ,7KBF,7TAN,3LZ1,3W96,4Z66,6R1U,6W5N,6J4Z,6M3V,6IR9,6SEF,7JO9,6R92,6T7D,1P34,5B1M,2F8N,7V9J,7PF6,3REL,6UPK,5KGF,3X1S,6J51,7JZV,6KIZ,3W97,6KXV,3W99,6WZ9,7YQK,7EGP,1P3K,5Z3U,6UGM,6J4W,1U35,5WCU,6KW3,6ESF,7OHB,5O9G,7MBM,3AZL,3KUY,6INQ,6YOV,5ONG,5B32,5Z23,6JR1,3MGR,7D69,3AZI,6MUP,7PEU,5HQ2,7V90,6VYP,6PWF,6XJD,6I84,6T79,5NL0,6J4Y,6MUO,7U4D,3A6N,1KX3,6UXW,7KBD,7PFF,6UPL,1EQZ,7DBH,6NQA,3AN2,5AV5,7XD0,4YS3,3REI,5DNN,3AZE,6JR0,6KIX,1P3A,6KW5,4LD9,7E8D,6T93,4J8W,5B33,6J4X,6GEN,7KBE,7A08,5Y0C,3AZJ,5CP6,1P3I,7PFW,7PII,1S32,7PFT,6G0L,6TDA,7SCZ,3X1V,1P3P,6L49,3O62,7PEY,5B1L,1P3B,1M18,6X59,4J8X,6V92,6M4D,7EG6,6VZ4,7V9S,7PFD,5B40,4YM6,7MBN,3MVD,7PFV,6UH5,6VEN,6W5I,6JM9,7BY0,3AZM,7PFU,7SCY,6R8Y,6BUZ,6A5L,5XM1,6LAB,1P3G,3LEL,6ESH,6T90,7VBM,3WKJ,3B6G,6OM3,7BXT,3MNN,7U53,7LYB,6HKT,6JXD,6LTJ,3MGP,5DNM,7CRO,6L9Z,3WTP,6A5O,7K5Y,3C1B,6RNY,7W9V,4J8V,5GT0,6NN6,6HTS,5GSE,6SE0,3AZH,6K1P,5XF6,7VDT,7NL0,5AY8,7OTQ,7U51,3UT9,6J99,5AV9,6SEG,6K1K,6IPU,4X23,6O1D,7CCR,6E0C,7PF3,7PF0,5XF3,6J50,7DBP,7VDV,6L4A,6Z6P,5GT3,1M19,1P3O,6ESI,6IQ4,2CV5,5AV8,6LER,7PEX,7XD1,6R8Z,6PWX,5OMX,3AZK,3MGQ,6T7C,5CPK,5B2J,7Y7I,5ONW,7WLR,5X0Y,5B24,1M1A,6M4G,7K5X,3AV1,7OHA,6ZHY,7PF2,7K7G,5B2I,5E5A,1ZLA,3X1U,6JYL,7K6Q,4YM5,7JOA,3AZN,7XCT,7VVU,7PEV,5AVC,7OHC,6T7B,6ZHX,6R25,3AV2,6ESG,7PFX,6PA7,6KIW,6SE6,6LA9,6FQ6,7SWY,1P3M,6R1T,3AZG,6LE9,7C0M,6KIU,7D1Z,5AV6,6PX3,1ID3,7EA8,7R5R,6RYU,6A5U,3TU4,6LA8,7PF5,7AT8,7K78,7E9C	441
canonical H2A	6E0P,3LZ0,7TN2,6PWE,5OXV,7PF4,6M44,6S01,5Z3L,5XF4,7CRR,6R93,6NJ9,6T7A,3REH,7ENN,6NOG,6R94,7K60,2NQB,6LA2,6W5M,6USJ,7PFA,3AYW,7U52,5GTC,1P3L,7U46,7K61,7CCQ,6DZT,6IY3,7BWD,6KW4,5B31,6IRO,7NKX,1ZBB,4WU8,4WU9,4Z5T,7CRP,6X5A,6Y5E,7NKY,2PYO,6T9L,6RYR,5JRG,3MGS,7XCR,5X7X,3UTB,6KE9,5OY7,6R91,7OH9,4R8P,6JMA,6L9H,1AOI,6R90,6V2K,6NZO,5Z3O,3W98,5B0Z,6SEE,4ZUX,6X0N,1KX4,7PFC,6KIV,5XF5,3REJ,3B6F,6Y5D,7U47,4QLC,6PWW,7XPX,7PFE,7V96,5MLU,5CPJ,6FTX,6NE3,6WZ5,6FQ5,6C0W,3UTA,6O96,6PWV,6R0C,3LJA,6A5R,5X0X,3AFA,2NZD,7V9K,2FJ7,7PEZ,7COW,5B0Y,6IY2,7VA4,6TEM,5CPI,1KX5,6FQ8,5Z3V,6PX1,4J8U,6A5P,5GXQ,1P3F,5F99,5AVB,4XZQ,3KWQ,5ZBX,7D20,7LYC,7K6P,7YYH,7CRQ,7LYA,7EA5,3KXB,4KGC,7PEW,7PET,7K63,7KTQ,5Y0D,3AZF,5XM0,7U50,6FML,3C1C,6KVD,7V9C,7VVZ,6A5T,3REK,7E8I,4XUJ,7KBF,7TAN,3LZ1,3W96,4Z66,6R1U,6W5N,6J4Z,6M3V,6IR9,6SEF,7JO9,6R92,6T7D,1P34,5B1M,2	407

	F8N,7V9J,7PF6,3REL,6UPK,5KGF,3X1S,6J51,7JZV,6KIZ,3W97,6KXV,3W99,6WZ9,7EGP,1P3K,5Z3U,6UGM,6J4W,5WCU,6KW3,6ESF,7OHB,5O9G,7MBM,3AZL,3KUY,6INQ,6YOV,5ONG,5B32,5Z23,6JR1,3MGR,3AZI,6MUP,7PEU,5HQ2,7V90,6VYP,6PWF,6XJD,6I84,6T79,5NL0,6J4Y,6MUO,7U4D,3A6N,1KX3,6UXW,7KBD,7PFF,6UPL,1EQZ,7DBH,6NQA,3AN2,5AV5,7XD0,4YS3,3REI,5DNN,3AZE,6JR0,6KIX,1P3A,6KW5,4LD9,7E8D,6T93,4J8W,6J4X,7KBE,7A08,5Y0C,3AZJ,5CP6,1P3I,7PFW,7PII,1S32,7PFT,6G0L,6TDA,7SCZ,3X1V,1P3P,6L49,3O62,7PEY,5B1L,1P3B,1M18,6X59,4J8X,6V92,7EG6,6VZ4,7V9S,7PFD,5B40,4YM6,7MBN,3MVD,7PFV,6UH5,6VEN,6W5I,6JM9,7BY0,3AZM,7PFU,7SCY,6R8Y,6BUZ,6A5L,5XM1,6LAB,1P3G,3LEL,6ESH,6T90,7VBM,3WKJ,3B6G,6OM3,7BXT,3MNN,7U53,7LYB,6HKT,6JXD,3MGP,5DNM,7CRO,6L9Z,3WTP,6A5O,7K5Y,3C1B,6RNY,7W9V,4J8V,6NN6,6HTS,5GSE,6SE0,3AZH,6K1P,5XF6,7VDT,7NL0,5AY8,7OTQ,7U51,3UT9,6J99,5AV9,6SEG,6IPU,4X23,6O1D,7CCR,6E0C,7PF3,7PF0,5XF3,6J50,7DBP,7VDV,6L4A,6Z6P,5GT3,1M19,1P3O,6ESI,6IQ4,2CV5,5AV8,6LER,7PEX,7XD1,6R8Z,6PWX,5OMX,3AZK,3MGQ,6T7C,5CPK,5B2J,7Y7I,5ONW,5X0Y,5B24,1M1A,7K5X,3AV1,7OHA,6ZHY,7PF2,5B2I,5E5A,1ZLA,6JYL,7K6Q,4YM5,7JOA,3AZN,7XCT,7VVU,7PEV,5AVC,7OHC,6T7B,6ZHX,6R25,3AV2,6ESG,7PFX,6PA7,6KIW,6SE6,6LA9,6FQ6,7SWY,1P3M,6R1T,3AZG,6LE9,7C0M,6KIU,7D1Z,5AV6,6PX3,7EA8,7R5R,6RYU,6A5U,3TU4,6LA8,7PF5,7AT8	
H2A.X	7WLR,6K1I,6GEJ,6LTJ,6K1J,6UPH,6QLD,6GEN,4JJN,7D69,7K7G,7ON1,1ID3,6K1K,4KUD,7YQK,7E9F,7K78,7E9C	19
H2A.Z	3WA9,5B32,1F66,6JOU,5B31,7M1X,3WAA,6M4D,5B33,5Z30	10
H2A.1	5GT0,3X1T,3X1U,5GSU	4
macroH2A	1U35,2F8N	2
H2A.B	6M4G,6M4H	2
canonical H2B	6E0P,3LZ0,7TN2,3WAA,6UPH,6PWE,5OXV,7PF4,6M44,6S01,5Z3L,5XF4,6M4H,7CRR,6R93,6NJ9,6T7A,3REH,7ENN,6NOG,6R94,5Z30,7K60,2NQB,6LA2,6W5M,6USJ,7PFA,3AYW,7U52,5GTC,1P3L,7U46,7K61,7CCQ,6DZT,6IY3,7BWD,6KW4,5B31,6IRO,7NKX,1ZBB,4WU8,4WU9,1F66,4Z5T,7CRP,6X5A,6Y5E,6JOU,7E9F,7NKY,2PYO,6T9L,6RYR,5JRG,6GEJ,3MGS,7XCR,5X7X,6K1J,3UTB,6KE9,5OY7,6R91,7OH9,4R8P,6JMA,6L9H,1AOI,6R90,6V2K,6NZO,4KUD,5Z3O,3W98,5B0Z,6SEE,4ZUX,6X0N,3WA9,1KX4,6K1I,7PFC,6KIV,5XF5,3REJ,3B6F,6QLD,4JJN,6Y5D,7U47,7M1X,4QLC,6PWW,7XPX,7PFE,7V96,5MLU,5CPJ,6FTX,6NE3,6WZ5,6FQ5,6C0W,3UTA,6O96,6PWV,6R0C,3LJA,6A5R,5X0X,3AFA,2NZD,7V9K,2FJ7,7PEZ,7COW,5B0Y,6IY2,7VA4,6TEM,5CPI,1KX5,6FQ8,5Z3V,6PX1,4J8U,6A5P,5GXQ,1P3F,5F99,5AVB,4XZQ,3KWQ,5ZBX,7D20,7LYC,7K6P,7YYH,7ON1,7CRQ,7LYA,7EA5,3KXB,4KGC,7PEW,7PET,7K63,7KTQ,5Y0D,3AZF,5XM0,7U50,6FML,3C1C,6KVD,7V9C,7VVZ,6A5T,3REK,7E8I,4XUJ,7KBF,7TAN,3LZ1,3W96,4Z66,6R1U,6W5N,6J4Z,6M3V,6IR9,6SEF,7JO9,6R92,6T7D,1P34,5B1M,2F8N,7V9J,7PF6,3REL,6UPK,5KGF,3X1S,6J51,7JZV,6KIZ,3W97,6KXV,3W99,6WZ9,7YQK,7EGP,1P3K,5Z3U,6UGM,6J4W,1U35,5WCU,6KW3,6ESF,7OHB,5O9G,7MBM,3AZL,3KUY,6INQ,6YOV,5ONG,5B32,5Z23,6JR1,3MGR,7D69,3AZI,6MUP,7PEU,5HQ2,7V90,6VYP,6PWF,6XJD,6I84,6T79,5NL0,6J4Y,6MUO,7U4D,3A6N,1KX3,6UXW,7KBD,7PFF,6UPL,1EQZ,7DBH,6NQA,3AN2,5AV5,7XD0,4YS3,3REI,5DNN,3AZE,6JR0,6KIX,1P3A,6KW5,4LD9,7E8D,6T93,4J8W,5B33,6J4X,6GEN,7KBE,7A08,5Y0C,3AZJ,5CP6,1P3I,7PFW,7PII,1S32,7PFT,6G0L,6TDA,7SCZ,1P3P,6L49,3O62,7PEY,5B1L,1P3B,1M18,6X59,4J8X,6V92,6M4D,7EG6,6VZ4,7V9S,7PFD,5B40,4YM6,7MBN,3MVD,7PFV,6UH5,6VEN,6W5I,6JM9,7BY0,3AZM,7PFU,7SCY,6R8Y,6BUZ,6A5L,5XM1,6LAB,1P3G,3LEL,6ESH,6T90,7VBM,3B6G,6OM3,7BXT,3MNN,7U53,7LYB,6HKT,6JXD,6LTJ,3MGP,5DNM,7CRO,6L9Z,3WTP,6A5O,7K5Y,3C1B,6RNY,7W9V,4J8V,5GT0,6NN6,6HTS,5GSE,6SE0,3AZH,6K1P,5XF6,7VDT,7NL0,5AY8,7OTQ,7U51,3UT9,6J99,5AV9,6SEG,6K1K,6IPU,4X23,6O1D,7CCR,6E0C,7PF3,7PF0,5XF3,6J50,7DBP,7VDV,6L4A,6Z6P,1M19,1P3O,6ESI,6IQ4,2CV5,5AV8,6LER,7PEX,7XD1,6R8Z,6PWX,5OMX,3AZK,3MGQ,6T7C,5CPK,5B2J,7Y7I,5ONW,7WLR,5X0Y,5B24,1M1A,6M4G,7K5X,3AV1,7OHA,6ZHY,7PF2,7K7G,5B2I,5E5A,1ZLA,3X1U,6JYL,7K6Q,4YM5,7JOA,3AZN,7XCT,7VVU,7PEV,5AVC,7OHC,6T7B,6ZHX,6R25,3AV2,6ESG,7PFX,6PA7,6KIW,6SE6,6LA9,6FQ6,7SWY,1P3M,6R1T,3AZG,6LE9,7C0M,6KIU,7D1Z,5AV6,6PX3,1ID3,7EA8,7R5R,6RYU,6A5U,3TU4,6LA8,7PF5,7AT8,7K78,7E9C	436

H2B.1	3X1V,5GSU,5GT3,3WKJ,3X1T	5
Human H1.4	7PFE,7PFD,7PFA,7PFC,7PEU,7PF0,7PEX,7PF2,7PEZ,7PET,7PF5,7PF6,7PF3,7PFT,7PFU,7PFX,7PFW,7PFV,7K5Y,7K63	20
Human H1.0	6LA2,6LAB,6LA8,7K5X,6LA9,7DBP,7COW	7
H5	4QLC,5WCU	2
Human H1.10	6L9Z,7K60	2
H1.8	7KBF	1
H1.0-B	5NL0	1
Widom 601 based DNA sequence	LZ0,3LZ1,3MVD,3TU4,4JJN,4LD9,4QLC,4R8P,4X23,4ZUX,5KGF,5MLU,5O9G,5OXV,5OY7,5WCU,5X0X,5X0Y,5Z3L,5Z3O,5Z3U,5Z3V,6BUZ,6C0W,6DZT,6E0C,6ESF,6ESG,6ESH,6ESI,6FML,6FQ5,6FQ6,6FQ8,6FTX,6G0L,6GEJ,6GEN,6HKT,6HTS,6I84,6IRO,6J99,6JYL,6K1P,6KIU,6KIV,6KIW,6KIX,6KIZ,6KW3,6KW4,6KW5,6L49,6L4A,6LTJ,6M4D,6M4G,6M4H,6NE3,6NJ9,6NN6,6NOG,6NQA,6NZO,6O96,6OM3,6PA7,6PWE,6PWF,6PWV,6PWW,6PWX,6PX1,6PX3,6QLD,6R1T,6R1U,6R25,6RYR,6RYU,6S01,6SE0,6SE6,6SEE,6SEF,6SEG,6T90,6T93,6T9L,6TDA,6TEM,6UH5,6UPH,6USJ,6UXW,6VEN,6VYP,6VZ4,6W5I,6W5M,6W5N,6WZ5,6WZ9,6X0N,6X59,6X5A,6XJD,6Y5D,6Y5E,6YOV,6Z6P,6ZHX,7A08,7AT8,7BWD,7BXT,7BY0,7C0M,7CCQ,7CCR,7CRO,7CRP,7CRQ,7CRR,7D1Z,7D20,7DBH,7DBP,7E8D,7E8I,7E9C,7E9F,7EA5,7EA8,7EG6,7EGP,7ENN,7JO9,7JOA,7JZV,7K5X,7K5Y,7K60,7K61,7K63,7K6P,7K6Q,7KBD,7KBE,7KTQ,7LYA,7LYB,7LYC,7M1X,7MBM,7MBN,7OH9,7OHA,7OHB,7OHC,7ON1,7OTQ,7PET,7PEU,7PEV,7PEW,7PEX,7PEY,7PEZ,7PF0,7PF2,7PF3,7PF4,7PF5,7PF6,7PFA,7PFC,7PFD,7PFE,7PFF,7PFT,7PFU,7PFV,7PFW,7PFX,7SCY,7SCZ,7TAN,7U4D,7U50,7U51,7U52,7VBM,7VDT,7VDV,7VVU,7W9V,7WLR,7XCR,7XCT,7XD0,7XD1,7Y7I,7YQK,6A5L,6A5O,6A5P,6A5R,6A5T,6A5U,6INQ,6IR9,6J4W,6J4X,6J4Y,6J4Z,6J50,6J51,6UPK,6UPL,6ZHY,7NKX 3UT9,5NL0,6K1K,6LAB,7D69,7KBF,7XPX 6JXD,6K1I,6L9Z,6LA2,6LA8,6LA9,6LER,6M3V,6M44,7COW,7SWY,7TN2,7U53,5HQ2,7K7G,7NKY	246
α-satellite based DNA sequence	1U35,2F8N,2NZD,3KUY,3MNN,3REH,3REI,3UTA,4J8U,4J8V,4J8W,4J8X,4KGC,4WU8,4WU9,4XUJ,5CP6,5DNM,5DNN,5XF3,5XF4,5XF5,5XF6,6IPU,6IQ4,6K1J 1AOI,1EQZ,1F66,1ID3,1KX3,1M18,1M19,1M1A,1P34,1P3A,1P3B,1P3F,1P3G,1P3I,1P3K,1P3L,1P3M,1P3O,1P3P,1S32,1ZLA,2CV5,2NQB,3A6N,3AFA,3AV1,3AV2,3AYW,3AZE,3AZF,3AZG,3AZH,3AZI,3AZJ,3AZK,3AZL,3AZM,3AZN,3C1B,3C1C,3KWQ,3KXB,3W96,3W97,3W98,3W99,3WA9,3WAA,3WKJ,3WTP,3X1S,3X1T,3X1U,3X1V,4KUD,4YM6,4Z5T,5AY8,5B0Y,5B0Z,5B1L,5B1M,5B24,5B31,5B32,5B33,5B40,5E5A,5GSU,5GT0,5GT3,5GTC,5GXQ,5X7X,5XM0,5XM1,5Y0C,5Y0D,5Z23,5Z30,5ZBX,6JM9,6JMA,6JOU,6JR0,6JR1,6KVD,6KXV,6V2K,6V92 1KX4,3REJ,3REK,3REL,3UTB 1KX5,1ZBB,2PYO,3B6F,3B6G,3LJA,3MGP,3MGQ,3MGR,3MGS,4XZQ,4YS3,4Z66,5AV5,5AV6,5AV8,5AV9,5AVB,5AVC,5OMX,5ONG,5ONW,7U46,7U47 6E0P,6MUO,6MUP,6O1D,7YYH,3O62,4YM5,5B2I,5B2J,6R93,6R94,6R8Y,6R8Z,6R90,6R91,6R92,7PII,7R5R,3AN2,3LEL,2FJ7	166
telomeric DNA sequence	6KE9,6L9H,6LE9,7V90,7V96,7V9C,7V9J,7V9K,7V9S,7VA4	10
MMTV DNA sequence	5F99,6IY2,6IY3	3

DNA1 DNA sequence	6T79,6T7A,6T7B,6T7C,6T7D	5
sat2l DNA sequence	5CPK	1
sat2R DNA sequence	5CPI,5CPJ	5
CEN3 DNA DNA sequence	7K78	1
Human-D02 DNA sequence	6R0C,6RNY	5
Other DNA DNA sequence	7NL0	1

Supplementary Table 2. PDB IDs for NCP complexes by molecular functions (from the figure 2).

**Molecular function category**	**PDB IDs**	**Number of structures**
PTM writers	6UGM,6X0N,6O96,6PWV,6KIV,7E8D,7XCR,7MBN,7VVZ,6UH5,6KIW,7CRO,7MBM,6VEN,6W5I,7BWD,6JM9,7W9V,7SCY,7LYC,7K6P,7CRR,7D20,7E8I,6NN6,4R8P,6JMA,6NJ9,6KIU,6NQA,7CRQ,7D1Z,6NOG,7JZV,6PWW,6KIZ,7CRP,7XPX,6PX3,5HQ2,7SCZ,7K6Q,6J99,7EA5,6NZO,7EA8,6PWX,7TAN,7LYB,7XCT,7VVU,6W5N,6W5M,7AT8,6KIX,6USJ<br><br>- Histone_methylation_H3K4_writer: 6UGM,6PWV,6KIV,7MBN,6UH5,6KIW,6VEN,7MBM,6W5I,6KIU,6PWW,6KIZ,6PWX,6W5N,6W5M,6KIX<br>- Histone_methylation_H3K79_writer: 7K6Q,6JMA,6J99,6NJ9,6O96,7BWD,6JM9,6NQA,7XCR,7XCT,6NOG,7K6P,6NN6<br>- Histone_methylation_H3K36_writer: 7EA5,6NZO,7EA8,7E8D,6PX3,7CRO,7CRR,7CRQ,7CRP<br>- Histone_methylation_H4K20_writer: 5HQ2,7XPX,7D1Z,7D20<br>- Histone_methylation_H3K27_writer: 7AT8<br>- Histone_ubiquitination_H2AK125_writer: 7JZV,7E8I,7LYC,7LYB<br>- Histone_ubiquitination_H2AK127_writer: 7JZV,7E8I,7LYC,7LYB<br>- Histone_ubiquitination_H2AK129_writer: 7JZV,7E8I,7LYC,7LYB<br>- Histone_ubiquitination_H2AK119_writer: 4R8P<br>- Histone_phosphorylation_H3T3_writer: 7TAN<br>- Histone_PARylation_writer: 6USJ,7SCY,6X0N,7SCZ	56

	- Histone_acetylation:<br>7VVZ,7W9V,7VVU	
Chromatin remodelers	6KW5,6RYR,7EG6,6VZ4,6GEJ,6KW3,6ZHX,7VDV,5X0Y,6LTJ,6FML,7TN2,5X0X,5O9G,6IY3,6GEN,6KW4,6UXW,5Z3L,6IRO,6ZHY,7NKX,6HTS,6IY2,6K1P,7VDT,6G0L,7ENN,7OTQ,6TDA,6JYL,6PWF,6FTX,6NE3,5Z3O,6RYU,5Z3V,6V92,7EGP,5Z3U<br><br>- CHD:<br>6RYR,6FTX,6ZHX,6RYU,6G0L,7ENN,7OTQ,7TN2,6ZHY,5O9G,7NKX<br>- INO80:<br>6HTS,6GEJ,6FML,6GEN<br>- ISWI:<br>6IRO,6PWF,6K1P,6NE3,6JYL<br>- SWI/SNF:<br>6KW5,6IY2,7EG6,6VZ4,6KW3,7VDV,5X0Y,7VDT,6V92,6LTJ,5X0X,6TDA,6IY3,6KW4,6UXW,5Z3L,5Z3V,5Z3O,7EGP,5Z3U<br><br>Human complexes:<br>- SWI/SNF (PBAF - 7VDV, 7VDT; cBAF - 6LTJ, there are no structures for ncBAF);<br>- CHD (ALC1- 6ZHX, 6ZHY, 7ENN, 7OTQ; CHD4 - 6RYU,6RYR; no structures for other CHD2-CHD9 complexes);<br>- INO80 (INO80 - 6HTS; no structures for SRCAP and TIP60);<br>- ISWI (SNF2h - 6NE3; no structures for SNF2L, hACF, NURF, CHRAC, NoRC, RSF, WICH)	40
Transcription factors	6IR9,6T7A,6J4Y,6J4W,6J50,6T7B,6J51,7OHB,6T90,6J4X,6S01,6YOV,6T7D,7OHA,6T7C,7OH9,6J4Z,7NKY,7NKX	19
Kinetochore components	6SEE,7K7G,7YYH,7PII,7ON1,6SEF,6MUP,6MUO,7U46,6FML,7U4D,6SE6,6QLD,7BXT,7R5R,4X23,7U47,6C0W	18
RNA polymerases	6IR9,6J4W,6J4Y,6J50,6A5L,6J51,6A5R,6J4X,6A5O,6INQ,6A5T,6A5U,6J4Z,6I84,6A5P,7NKY,7NKX	17
cGAS	7JOA,7C0M,7JO9,6X5A,6Y5E,7A08,6X59,6Y5D,7CCQ,6XJD,7CCR	11
PTM erasers	6R25,4ZUX,6Z6P,6T9L,6R1U,6VYP	6
PTM readers	6R25,5KGF,6R1U,6S01,6JMA,6VEN,6FTX,6J99<br>- Histone_ubiquitination_H2BK120_recognition: 6JMA,6VEN,6FTX,6J99	8
Histone chaperones	6UPL,7NKY,6UPK	3
DNA integrations components	6RNY,5MLU	2
Histone exchange	6GEJ,6GEN	2
DNA methyl-transferases	6PA7	1

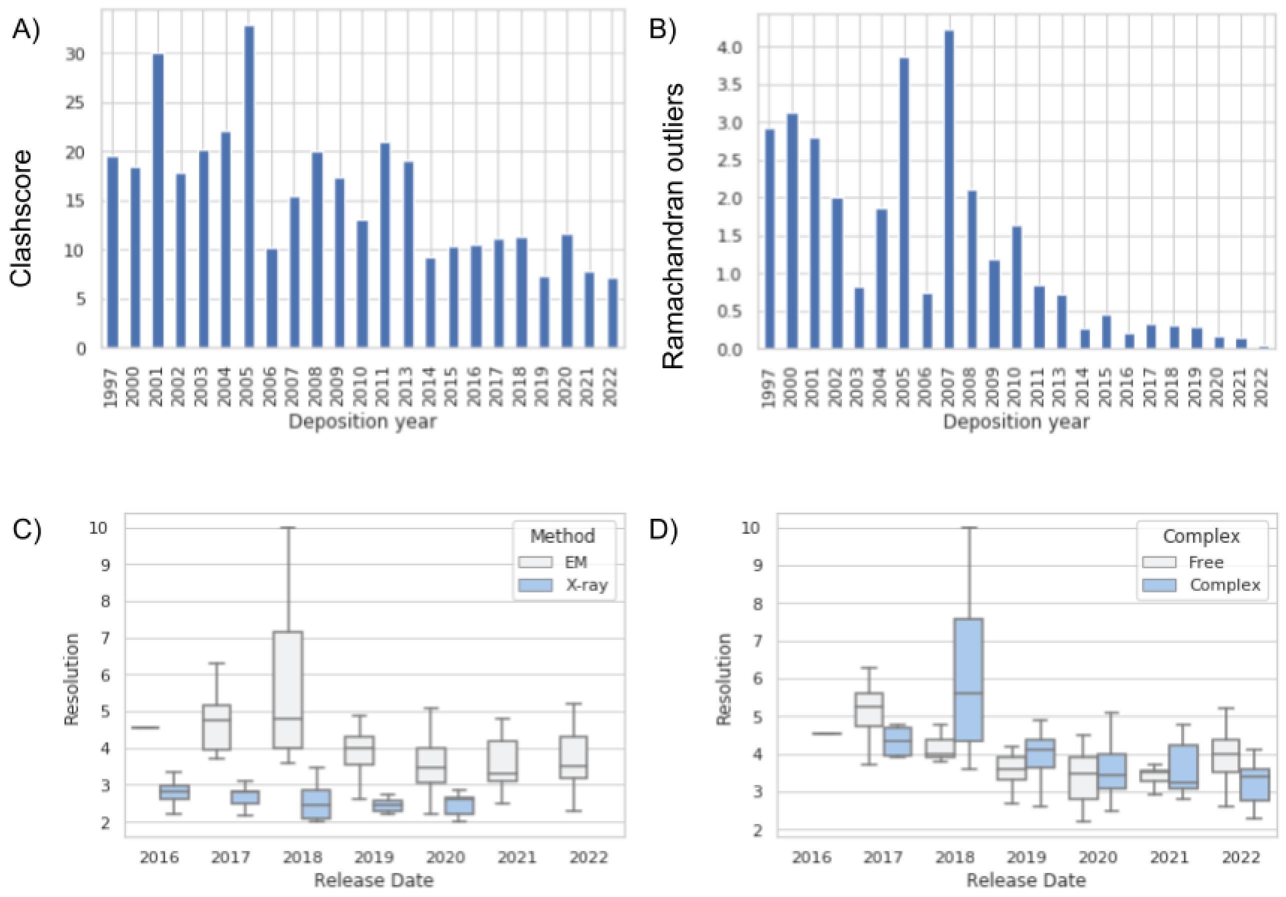

Supplementary Figure 1. A) Average per-year clashscore. B) Average per year amount of ramachandran outliers. C) Distribution of resolutions of nucleosome structures containing single nucleosome obtained by different methods D) Distribution of resolutions of nucleosome structures containing single nucleosome or nucleosome with interacting proteins.

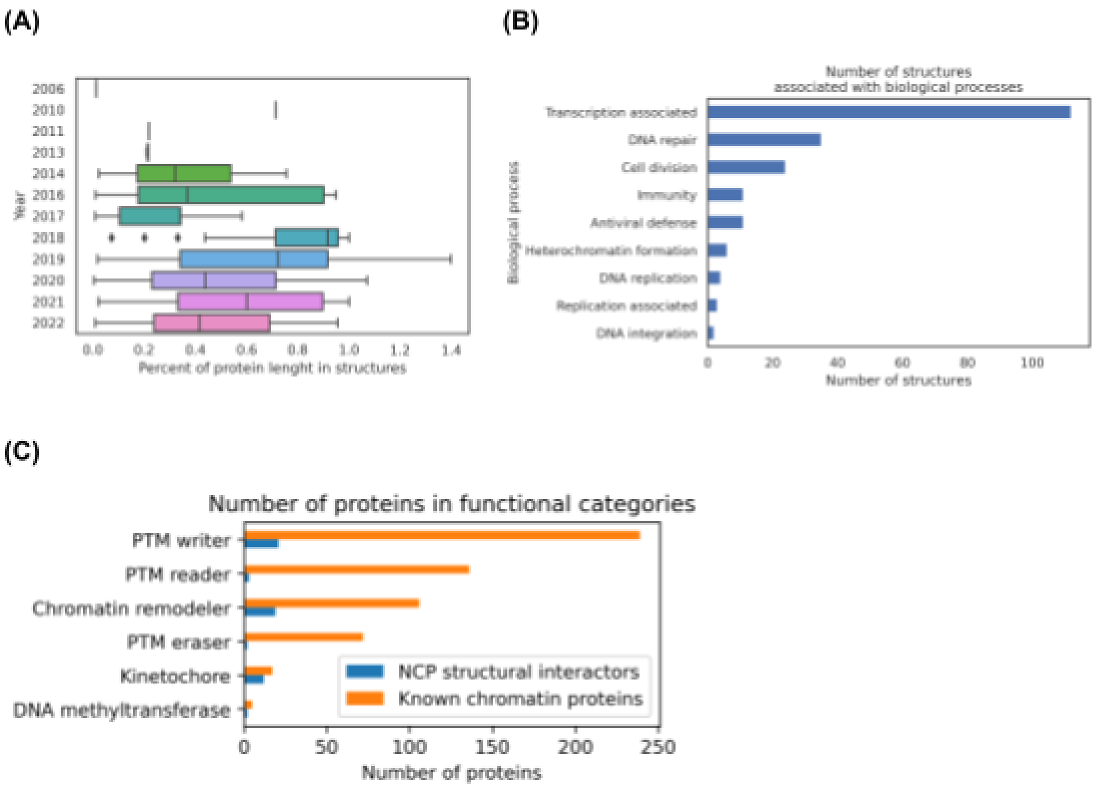

Supplementary Figure 2. (A) The coverage of non-histone protein sequences in NCP complex structures (in comparison with full protein sequences). The value more than 1 indicates fusion-proteins. (B) Number of structures NCP in complexes associated with biological processes. (C) The coverage of known human chromatin proteins by NCP interactors in different functional categories.
